# PLXNB1/SEMA4D signals mediate interactions between malignant epithelial and immune cells to promote colorectal cancer liver metastasis

**DOI:** 10.1111/jcmm.70142

**Published:** 2024-10-23

**Authors:** Zixue Xuan, Yuan Zhang, Dan Li, Kai Wang, Ping Huang, Jiana Shi

**Affiliations:** ^1^ Center for Clinical Pharmacy, Cancer Center, Department of Pharmacy Zhejiang Provincial People's Hospital (Affiliated People's Hospital), Hangzhou Medical College Zhejiang Hangzhou China; ^2^ Department of Pharmacy Zhejiang Provincial People's Hospital Bijie Hospital Bijie Guizhou China; ^3^ Key Laboratory of Epigenetics and Oncology, Research Center for Preclinical Medicine Southwest Medical University Luzhou Sichuan China; ^4^ Key Laboratory of Endocrine Gland Diseases of Zhejiang Province Zhejiang Provincial People's Hospital (Affiliated People's Hospital), Hangzhou Medical College Zhejiang Hangzhou China

**Keywords:** colorectal cancer, liver metastasis, PLXNB1/SEMA4D, single‐cell sequencing, tumour microenvironment

## Abstract

Distal metastases result from metastatic microenvironment and tumour epithelial cell interactions, the cellular heterogeneity of primary colorectal cancer (CRC) and liver metastases (LM) was evaluated by integrating single‐cell sequencing data, and the collected gene expression data from metastatic epithelial cell subsets was used to construct a prognostic model and to identify intercellular receptor‐ligand interactions between epithelial and immune cells in CRC and LM. Multiplex immunofluorescence staining, and in vitro wound healing, cell migration and cell apoptosis assays were performed to further explore the biological relevance of identified potential regulatory molecules. In this study, approximately 17 epithelial cell subtypes were detected, with Epi‐11 cells being highly expressed in LM tissues compared with CRC samples. Furthermore, patients with high expression of the metastasis‐related genetic profile of Epi‐11 had a poorer prognosis. By predicting receptor–ligand interactions, Epi‐11 cells were found to interact more with myeloid and T/natural killer cells in LM tissues when compared to primary CRC samples, which was mediated by the PLXNB1/SEMA4D axis. In addition, high *SEMA4D* expression was correlated with decreased overall survival of patients with CRC, whereas *PLXNB1* was not. *SEMA4D* knockdown prevented the migration and promoted the apoptosis of HCT116 cells in vitro. In summary, Epi‐11 cells, an important subset of epithelial cells, may drive the LM of CRC and act by crosstalk with immune cells through the PLXNB1/SEMA4D signalling axis.

## 1 INTRODUCTION

Approximately 15%–25% of patients with colorectal cancer (CRC) have liver metastasis (LM) at diagnosis, and 25% of patients develop LM after radical resection of the primary CRC.^1^ Importantly, these patients often have a poor prognosis, with a 5‐year survival rate of only 14%; therefore, a deeper understanding of LM is detrimental to improve CRC outcomes. In recent years, several advances have been made with regards to the underlying mechanisms of CRC‐derived LMs.^2^, ^3^, ^4^ Numerous studies have shown that genetic abnormalities,^5^ tumour cell heterogeneity,^6^ epithelial–mesenchymal transformation,^7^ tumour microenvironment and other factors are involved in this metastatic process.^8^ Previous studies have revealed the presence of various cell types in the tumour microenvironment, such as cancer‐associated fibroblasts, tumour‐associated macrophages and different immune cell subsets, the specific roles of these cell subsets in CRC‐associated LMs and their interactions with tumour cells remain unclear.^9^, ^10^ Recent findings suggest that identifying the tumour cell subpopulations that drive metastasis is crucial for preventing cancer spread and recurrence.^11^, ^12^ Of note, cancer epithelial cells were identified as one of the culprits driving LMs in CRC^13^, ^14^; however, the mechanisms by which epithelial cells drive LMs in CRC remains poorly understood. For example, Wang R et al. found that tumour epithelial cells generally exhibit stem/progenitor cell markers and possess mesenchymal‐like characteristics, while their subclones exhibited severe copy number variations (CNVs) and had the potential to metastasize to distant organs.^15^ Another study identified an intrinsic‐consensus of the molecular subtypes of CRC epithelial cells (identified as iCMS2) that had higher WNT and MYC activation, as well as higher CNV levels.^16^ Additionally, a report by Cañellas‐Socias et al. showed that a subpopulation of epithelial cells expressing EMP1 can enrich the liver after surgery to remove CRC tumours, thereby leading to LM metastasis and cancer recurrence. Indeed, targeting EMP1^+^ cells effectively prevented tumour recurrence after surgery in preclinical models.^17^ Therefore, techniques such as single‐cell sequencing and multi‐omics approaches at various levels can help us better understand the metastatic process of CRC and identify potential therapeutic targets.^18^, ^19^, ^20^ To gather additional information on how CRC‐associated epithelial cells can drive LMs, we investigated the cellular heterogeneity of primary CRC and LM by integrating single‐cell data and analysed the intercellular receptor‐ligand interactions between these epithelial and immune cells in the tumour microenvironment. Interestingly, our results showed that Epi‐11, a subtype of malignant epithelial cells, may play an important role in LM development in CRC by mediating interactions with immune cells via the PLXNB1–SEMA4D axis.

## 2 METHODS

### 2.1 Single‐cell sequencing data collection and analysis

Single‐cell sequencing (scRNA‐seq) data from a total of eight primary CRC and paired LM tissues were obtained from two publicly available datasets (accession number: GSE178318 [samples GSM5387665–70] and GSE225857 [samples CNS0502040, CNS0502042, CNS0502044–46]) (Table S1).^21^, ^22^ We used the Seurat R package (v4.1.1) to integrate two datasets of single‐cell sequencing data (dims = 1:50). Batch effect correction was performed using Harmony and clustering of major cell types was analysed using the *FindNeighbors* function in Seurat (reduction = ‘harmony,’ dims = 1:25) and *FindClusters* (resolution = 0.5), respectively. Cluster visualization was performed using *RunUMAP* (reduction = ’harmony,’ dims = 1:50). We used the FindAllMarkers function in Seurat to conduct differential gene expression analysis between cell types or clusters, with the following parameters: (1) differential genes with >0.25‐fold difference (log‐scale) on average between the two groups of cells and detectable expression in more than 25% of cells in either of the two populations. (2) P value <0.05 (Wilcoxon Rank Sum test).^22^ When annotating epithelial cell subtypes, we determined dims = 1:20, resolution = 0.5 for subpopulation analysis based on the results of the Elbow plot. Epithelial cell subsets were preliminarily determined by comparing the proportion of metastatic epithelial cell subgroups in CRC and LM tissues with that of cancer cells.^22^

### 2.2 Identification of epithelial cell subsets driving metastasis

The R package *inferCNV* (https://github.com/broadinstitute/inferCNV; v1.2.1) was used to analyse the copy number instability as proxy of copy number alterations from cancer scRNA‐seq data.^23^ Moreover, *inferCNV* was used to compare the gene expression profile of malignant cells with that of healthy cells (B cells were used as reference). In addition, mitochondrial chromosomes and sex chromosomes were excluded from the analysis. Finally, the *ssgsea* and *singscore* methods in the R package *irGSEA* were used to determine the gene enrichment score matrixes of each subpopulation and to identify the possible epithelial cell subsets driving metastasis.^25^

### 2.3 Construction of a prognostic model based on differentially expressed genes (DEG) of metastatic epithelial cell subsets

Clinical and genetic data from CRC patients was obtained from The Cancer Genome Atlas (TCGA) database. All samples with incomplete prognostic information and follow‐up data for <7 days were removed, and the DEG among patients with versus without metastases, as well as the characteristic genes of the metastatic epithelial cell subsets mentioned above, were screened. *ssgsea* was used to score the identified characteristic gene set of epithelial cells related to metastasis and the R package *maxstat* (v0.7–25) was used to define its optimal cut‐off value, which was used to divide the patients into high‐ and low‐risk groups.^26^ The minimum and maximum sample size was set at >25% and <75%, respectively. The Survfit function of the R software package *survival* was used to analyse the differences in prognosis between the two groups.^27^ The log‐rank test was used to evaluate significant prognostic differences among the groups.^28^

### 2.4 Cell‐to‐cell communication assessment

Cell‐to‐cell interactions were evaluated based on the expression of receptor‐ligand pairs in specific cell populations. Specifically, the interaction score was established based on the average expression values of each ligand‐receptor partner in the corresponding pair of cells. Finally, *CellPhoneDB* was used to analyse intercellular receptor‐ligand interactions between epithelial and immune cells in CRC and LM samples.^29^

### 2.5 Multiplex immunofluorescence assay

A tissue microarray cohort (HColA160CD01) was purchased from Shanghai Outdo Biotech Company (Shanghai, China). The study was conducted in strict adherence to the principles outlined in the Declarations of Helsinki and Istanbul. It received approval from the Ethics Committee of Shanghai Outdo Biotech Company, under the reference number SHXC2021YF01. Multiplex immunofluorescence staining of formalin‐fixed paraffin‐embedded tissue sections of CRC, LM and adjacent normal tissue (ANTs) was performed using a three‐colour multi‐labeling kit (abs50028; Absin Biosciences, Shanghai, China). Different fluorescence signals from Opal 620 and 690 nm were generated, corresponding to CD8 and SEMA4D (dilution: 1:2000 and 1:200; ab245118 and ab307685; Abcam, Cambridge, UK), respectively. The sections were counterstained with DAPI for nuclear visualization and analysed.^30^

### 2.6 Wound healing, cell migration and cell apoptosis assays

HCT116 cells were transfected with *SEMA4D*‐targeting or non‐targeting small interfering RNAs and cultured for 24 h. These cells (6 × 10^5^) were then seeded into 6‐well plates, the cell layer was disrupted using a 10 μL pipette tip; after incubation for 48 h, the cells were rinsed thrice with phosphate buffered saline and observed under an inverted microscope. For the migration assay, the cells (6.5 × 10^4^) were seeded in serum‐free medium in the upper chamber of a transwell plate and cell medium containing 10% serum was added to the lower chamber; after 48 h of incubation, the cells were fixed, stained and photographed. To assess cell viability, transfected cells (3 × 10^5^) were seeded into 6well plates and subsequently resuspended in 500 μL of binding buffer supplemented with Annexin V‐FITC and propidium iodide after 24 h of culture, according to the manufacturer's instruction; the number of apoptotic cells was determined by flow cytometry.^31^

### 2.7 Statistical analysis

One‐way analysis of variance or Student's *t*‐test were used to compare two groups. The results are presented as mean ± standard error of the mean (SEM). Statistical significance was set at *p* < 0.05.

## 3 RESULTS

### 3.1 Differences in cell subsets between CRC and LM tissues

**FIGURE 1:**
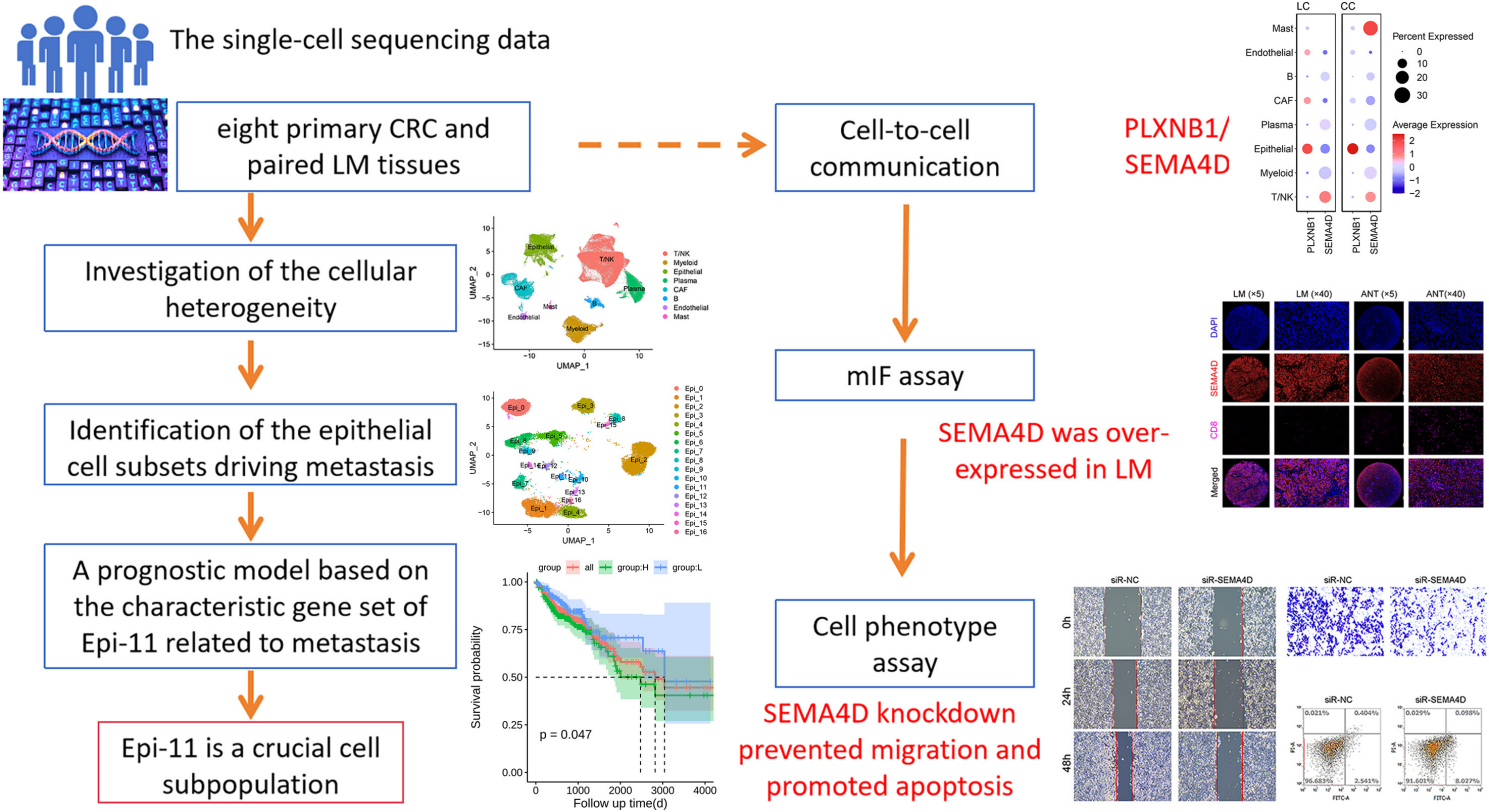
Detailed workflow of our study.

**FIGURE 2:**
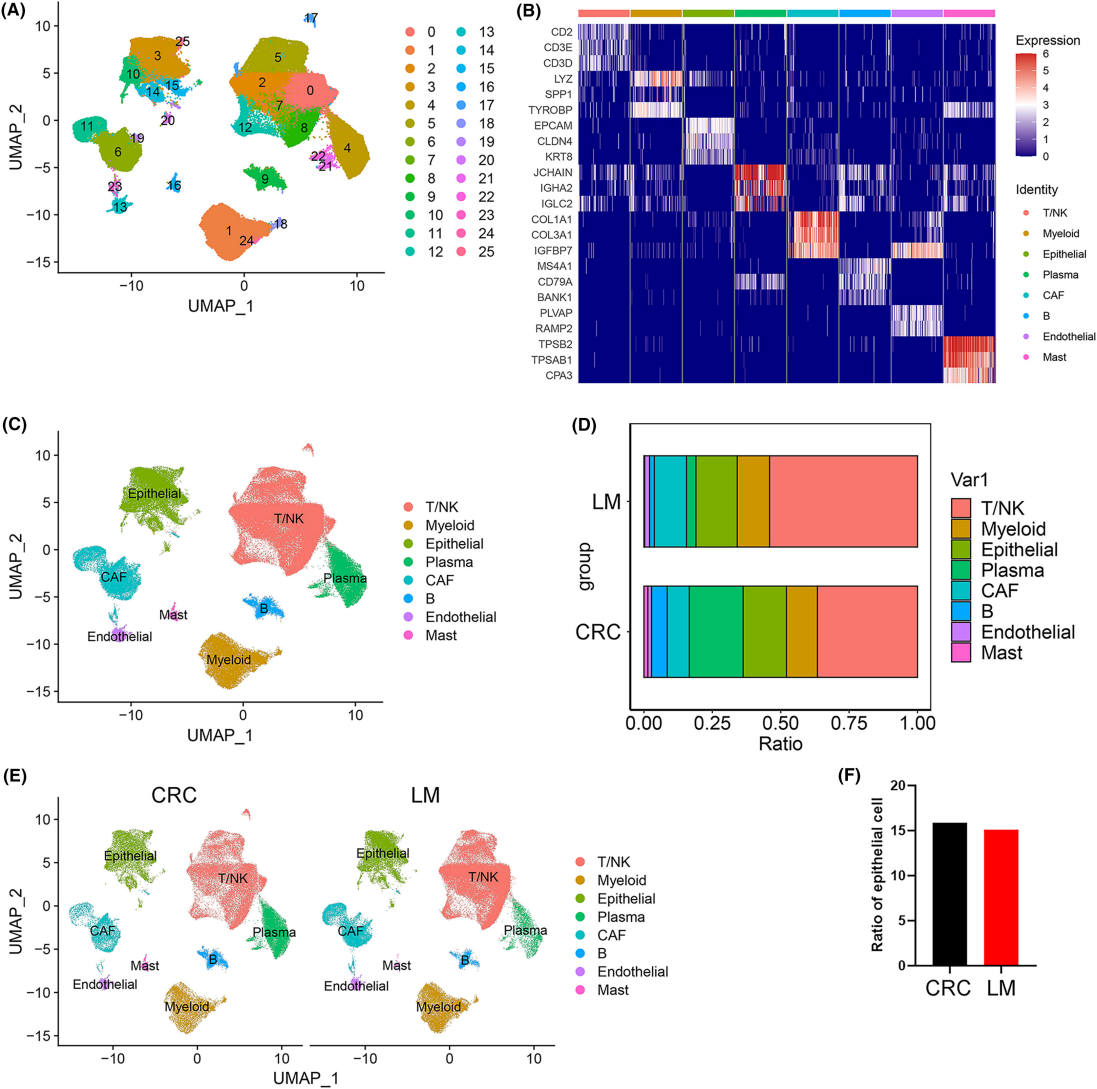
Single‐cell transcriptional profiling of colorectal cancer (CRC) and liver metastasis (LM) tissues. (A) Uniform manifold approximation and projection (UMAP) of 26 major clusters. (B) The markers of the cell type. (C) UMAP of eight cell types. (D) The proportions of these eight cell types in CRC and LM tissues. (E) UMAP of cell types in CRC and LM tissues. (F) The difference in the proportion of epithelial cells between the CRC and LM tissues.

The detailed workflow of our study is presented in Figure 1. To explore the heterogeneity of CRC and LM tissues at the single‐cell level, we evaluated scRNA‐seq data from two publicly available datasets. The cells were clustered according to marker genes (Table S2) and cell types were defined according to marker genes (Table S3). Notably, the cells from the 16 analysed samples clustered into 26 major clusters, which were further explored by principal component analysis and visualized using the uniform manifold approximation and projection (UMAP) approach (Figure 2A; Figure S1). The cell type of each cluster was annotated using well‐established gene markers (Figure 2B); specifically, epithelial (3, 10, 14, 15, 20, 25 cluster), endothelial (13 cluster), myeloid (1, 18, 24 cluster), T/natural killer (NK) (0, 2, 5, 7, 8, 12, 17 cluster), B (nine cluster), mast (16 cluster) and plasma (4, 21, 22 cluster) cells, as well as cancer‐associated fibroblasts (6, 11, 19, 23 cluster) were identified (Figure 2C). A comparison of the frequency of these eight cell types in CRC and LM tissues (Figure 2D) revealed no significant differences between the two groups relating to the proportion of epithelial cells (Figure 2E,F).

**FIGURE 3:**
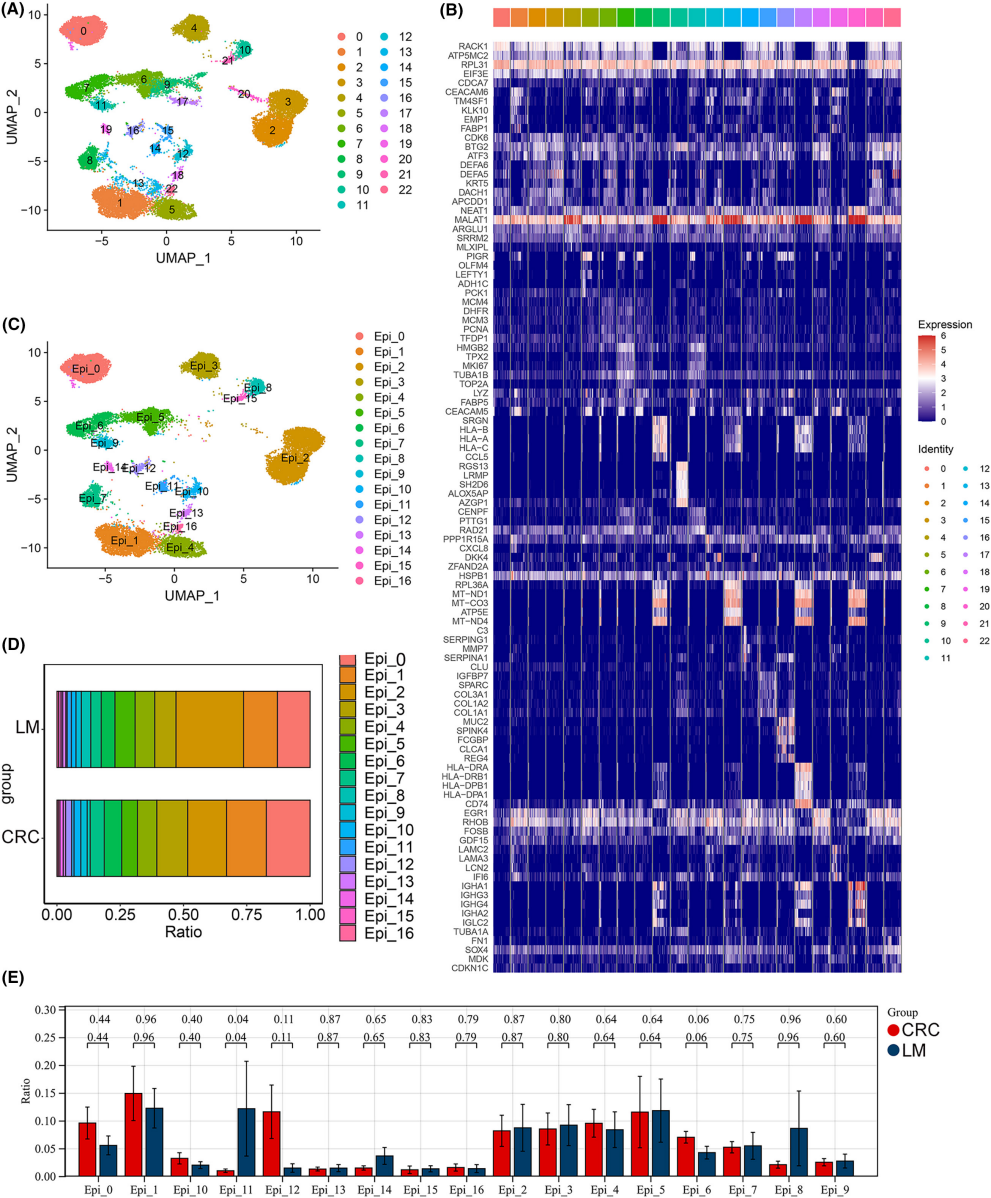
Single‐cell RNA‐seq analysis reveals epithelial subpopulations in CRC and LM tissues. (A) The UMAP of epithelial cell subclusters. (B) Heatmap showing the expression levels of top marker genes in epithelial cell subclusters. (C) UMAP of 17 subtypes in epithelial cells. (D, E) The proportions of subtypes in epithelial cells of CRC and LM tissues.

**FIGURE 4:**
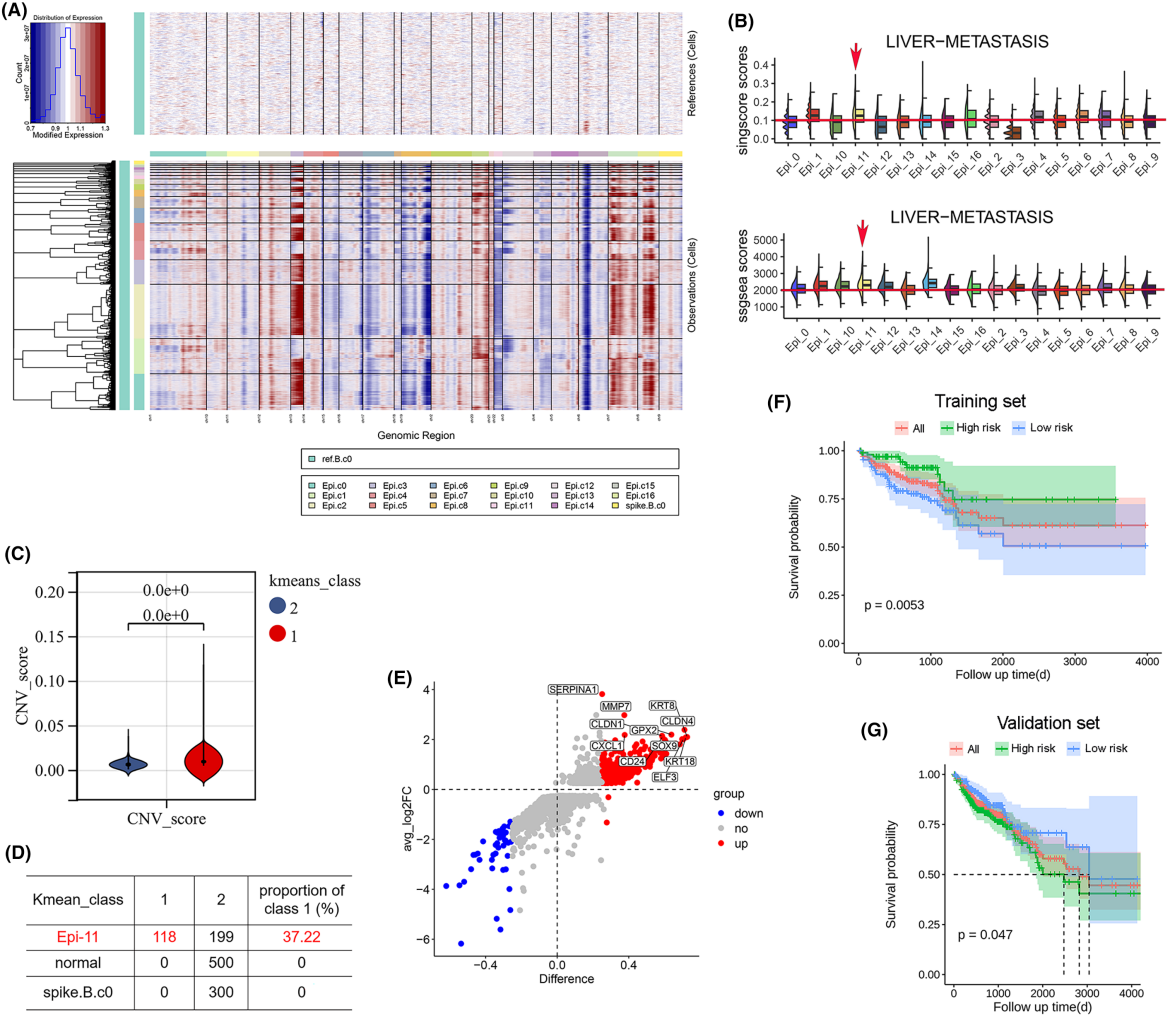
Screening of epithelial cells driving LM in CRC. (A) InferCNV's analysis. (B) The ssgsea and singscore scores of subtypes in epithelial cells. (C) Each Epi subtype was clustered into one of two classes. (D) Cell distribution in two classes of Epi‐11. (E) The differentially expressed genes (DEGs) between patients with metastasis and those without metastasis in The Cancer Genome Atlas (TCGA). (F) A prognostic model was constructed using a training set containing 214 samples. (G) A prognostic model was constructed using a validation set.

Next, we explored and compared the features of the epithelial cell populations present in CRC and LM tissues. Sub classification of these epithelial cells revealed 23 clusters that were present in all patients (Figure 3A), which corresponded to 17 cell subtypes (as determined by several gene markers) (Figure 3B,C). The epithelial cell grouping marker is displayed in Table S4. Of note, Epi‐11 cells were observed more frequently in LM than in CRC tissues (Figure 3D,E). Furthermore, InferCNV analysis revealed that several epithelial cell subtypes had high CNV scores (Figure 4A), whereas the gene expression profile of the epithelial cell subtypes revealed that the NABA_MATRISOME_METASTATIC_COLORECTAL_LIVER_METASTASIS gene set was significantly higher in Epi‐11 cells (Figure 4B; Table S5,S6). InferCNV analysis indicated that each epithelial cell subtype was clustered into two classes, the reference cells all clustered in class 2, while the cells in class 1 may be cancer cells (Figure 4C). The distribution of Epi‐11 cells within the two classes is shown in Figure 4D, indicating that Epi‐11 contained highly malignant tumour cells. Taken together, these results suggest that Epi‐11 may be a crucial cell subpopulation that drives metastasis in CRC.

### 3.2 Construction of a prognostic model based on the characteristic gene set of Epi‐11 related to metastasis

To further explore the impact of Epi‐11 cells on the overall disease progression in patients with CRC, we needed to identify the specific genes involved in this process. To do so, we used genetic and clinical data of 395 CRC patients that were publicly available in the TCGA database and compared the genetic profile of non‐metastatic versus metastatic cases (*n* = 331 and 64, respectively). The DEGs between patients with and without metastases in the TCGA dataset are shown in Figure 4E. Next, the characteristic genes of Epi‐11 cells that were related to metastasis (log[FoldChange] >1.0, adjusted *p* <0.05) were identified as the intersection of the Epi‐11 scRNA‐seq (Table S7) and non‐metastatic versus metastatic TCGA (Table S8) datasets. Lastly, the metastatic Epi gene set was identified (Table S9), and patients were divided into high and low risk groups (best cut‐off value: 5.286) based on the TCGA clinical information (Table S10). This approach allowed to design a CRC prognostic model, which confirmed that the presence of Epi‐11 cells within the tumour microenvironment can effectively help predict the risk of metastasis and overall survival of patients with CRC (Figure 4F).

### 3.3 Aberrant PLXNB1/SEMA4D interaction contributes for LM development in CRC

**FIGURE 5:**
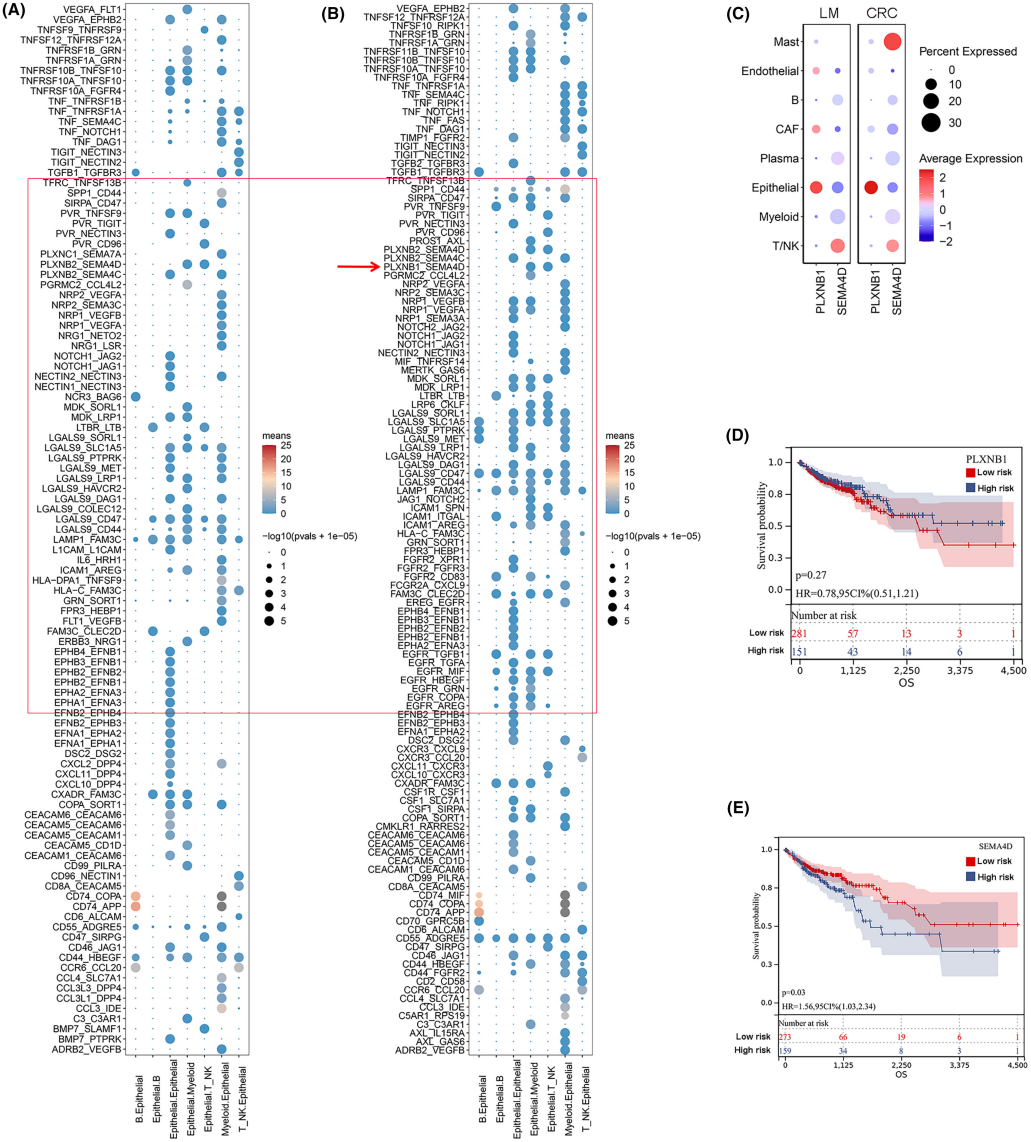
The aberrant interaction of PLXNB1‐SEMA4D occurs in LM of CRC. (A) Receptor‐ligand interactions between cell types in CRC. (B) Receptor‐ligand interactions between cell types in LM. (C) The expression of PLXNB1 and SEMA4D in different cell types of CRC and LM tissues. (D) Correlation between PLXNB1 expression level and overall survival using TCGA data. (E) Correlation between SEMA4D expression level and overall survival using TCGA data.

**FIGURE 6:**
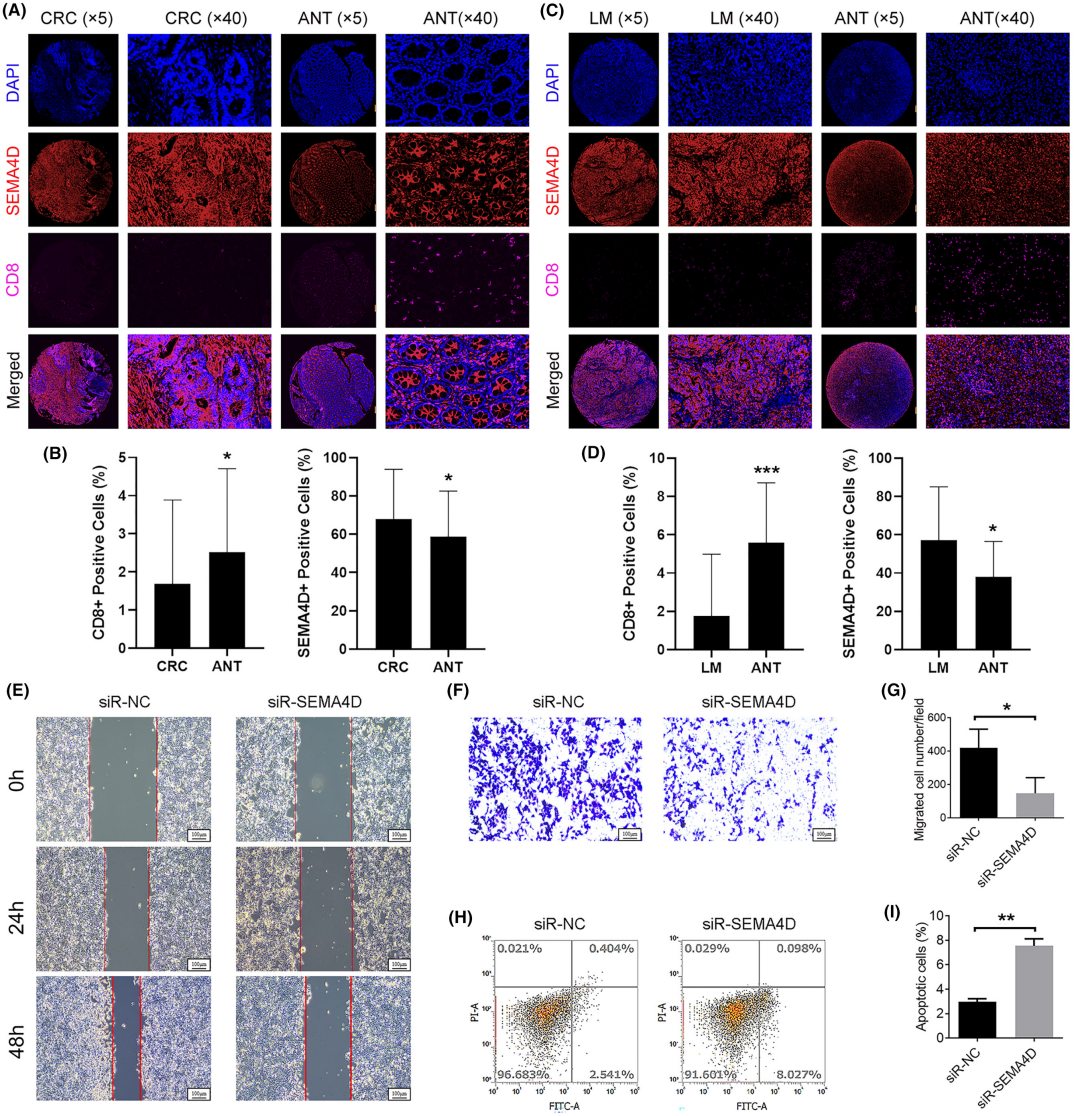
SEMA4D is required for human CRC cell migration and growth. (A, B) SEMA4D was overexpressed and CD8 was underexpressed in CRC, compared to ANTs. (C, D) SEMA4D was overexpressed and CD8 was underexpressed in LM, compared to ANTs. (E) The wound healing assay showed that SEMA4D knockdown inhibited HCT116 cell migration. (F, G) The cell migration assay showed that SEMA4D knockdown inhibited HCT116 cell migration. (H, I) SEMA4D knockdown promoted cell apoptosis.

To better understand how Epi‐11 cells mediate LM development, we predicted cell‐to‐cell communication mechanisms that could occur in LM and CRC tumours via receptor‐ligand interactions. Overall, LM cells seemed to potentially establish more interactions than cells within the primary CRC tumours, particularly between Epi‐11 and myeloid or T/NK cells. Of note, the PLXNB1/SEMA4D pair appeared specifically in the LMs (Figure 5A,B), with *PLXNB1* and *SEMA4D* being predominantly expressed in epithelial cells and in mast and T/NK cells, respectively (Figure 5C). *PLXNB1* expression was not found to be significantly associated with the overall survival of CRC patients (Figure 5D, Figure S2), whereas patients with high *SEMA4D* expression had a significantly poorer survival rate (Figure 5E; Figure S2). These findings suggest that *PLXNB1* does not play a direct role but may be involved in the regulation of tumour progression through its interaction with *SEMA4D*. Immunofluorescence analysis of LM and CRC tissues from 36 patients (Table S11) further confirmed that SEMA4D was significantly overexpressed in LM and CRC tissues compared with healthy colon tissue samples; additionally, CD8 was significantly downregulated in these cancer samples (Figure 6A–D).

### 3.4 SEMA4D is required for CRC cell migration and growth

To assess the role of SEMA4D in human CRC metastasis, we performed in vitro wound healing and cell migration assays. Notably, SEMA4D knockdown inhibited HCT116 cell migration (Figure 6E–G). In addition, SEMA4D knockdown promoted cell apoptosis (Figure 6H,I). These results suggest that SEMA4D contributes to the development of CRC metastases and cell growth.

## 4 DISCUSSION

The cell composition of solid tumours is highly heterogeneous, comprising epithelial and immune cells, among others, which jointly shapes tumorigenesis, tumour development and overall outcome.^32^ During the process of LM in CRC, the role of epithelial cells is multifaceted, involving complex interactions within the tumour microenvironment and the heterogeneity of tumour cells.^33^ For instance, epithelial cells acquire mesenchymal properties through the EMT process, enhancing their invasive and metastatic capabilities.^34^ Studies have found that tumour epithelial cells expressing specific markers (such as SOX9 and MKI67) exhibit stem cell‐like characteristics, possessing the potential for self‐renewal and differentiation, and may play a key role in the occurrence, development and metastasis of cancer.^15^ Additionally, the interaction between tumour epithelial cells and immune cells significantly impacts immune escape and progression of the tumour. For example, tumour cells may evade immune surveillance by affecting the infiltration and function of T cells.^8^ Research has revealed the presence of heterogeneous cancer stem cells in CRC and their organ‐specific metastasis, indicating that certain tumour epithelial cells may have a propensity to metastasize to specific organs (such as the liver or ovaries).^2^ LM development in CRC is associated with poor prognosis; therefore, it is of great biological and clinical significance to study the interaction between cancer epithelial and immune cells. In this study, we analysed single‐cell data of epithelial cells associated with LM in CRC and found that Epi‐11 cells, which are more frequently observed in LM tissues, are associated with a high degree of malignancy. Indeed, we designed a prognostic model based on the metastasis‐related genetic profile of the Epi‐11 cell subpopulation that could accurately predict the prognosis of patients with CRC. Cell–cell interactions are critical for various biological processes, including tissue organization, immune response and cellular signalling. These interactions can occur at different levels, ranging from intracellular events to extracellular conditions,^35^ and play a significant role in tumour metastasis.^36^ Therefore, by predicting the receptor‐ligand interactions and calculating the interactions between Epi‐11 and immune cells, we found that Epi‐11 is more likely to interact with myeloid and T/NK cells in LM than in CRC tissues. Importantly, our results revealed that an aberrant PLXNB1/SEMA4D interaction occurs only in LMs, with *PLXNB1* and *SEMA4D* being predominantly expressed in epithelial and mast and T/NK cells, respectively. Notably, while *PLXNB1* was not associated with the overall survival of patients with CRC, high *SEMA4D* expression was associated with significantly decreased overall survival, suggesting that Epi‐11 may not be directly involved in tumorigenesis through *PLXNB1*, but may promote LM development by interacting with *SEMA4D* on mast and T/NK cells. SEMA4D, serving as a pleiotropic signalling protein, is implicated in tumour growth and metastasis, hence why the elevated expression levels of SEMA4D are significantly correlated with patient survival. PLXNB1, acting as the high‐affinity receptor for SEMA4D, might exert a more intricate function within the tumour microenvironment. The expression levels of PLXNB1 could be subject to a multitude of influences, such as the composition of the tumour microenvironment and crosstalk with other signalling pathways. These factors might account for the lack of significant association between PLXNB1 expression levels and patient survival outcomes. However, the role of the PLXNB1/SEMA4D axis in tumours has been elucidated by several studies. For instance, PLXNB1/SEMA4D promotes metastasis of head and neck squamous cell carcinoma by inducing epithelial–mesenchymal transition.^37^ Moreover, SEMA4D expression was shown to contribute to the development of bone metastases in lung cancer,^38^ with its inhibition preventing the growth of various cancers in vivo.^39^ In CRC, the PLXNB1/SEMA4D signals were found to induce angiogenesis and aggressive growth of CRC, and may be useful tools in predicting disease recurrence in patients with CRC.^40^ Therefore, the PLXNB1/SEMA4D axis may be a relevant cancer biomarker that warrants further investigation, with SEMA4D's specific role in CRC remaining unclear. Interestingly, the present study provides in vitro evidence that SEMA4D is involved in tumour progression and metastasis in CRC, and that its inhibition prevents the invasion and migration of HCT116 cells, which corresponds to the findings of previous reports.

## 5 CONCLUSION

In summary, this study provides new insights into the role of the PLXNB1/SEMA4D axis in LM development of CRC, as well as providing a more comprehensive understanding of the immunological characteristics of tumour metastasis. In addition, the scRNA‐seq data analysed herein reveals new key regulatory factors that are involved in the mutual selection process of cancer epithelial and immune cells, thereby revealing potential therapeutic targets and molecular markers for LM in CRC. Despite these relevant findings, this study has some limitations. First, the prognostic model described herein should be validated using a larger clinical cohort. Additionally, in‐depth studies on how PLXNB1/SEMA4D signals meditate the epithelial‐immune cells interaction to promote LM in CRC should be performed. Taken together, this study demonstrates that LM development in CRC does not result from the unilateral action of cancer epithelial or immune cells, but from a complex dialogue among cells.

## AUTHOR CONTRIBUTIONS

**Zixue Xuan:** Data curation (equal); funding acquisition (equal); investigation (equal); methodology (equal); visualization (equal); writing – original draft (equal). **Yuan Zhang:** Data curation (equal); methodology (equal); writing – original draft (equal). **Dan Li:** Data curation (equal); writing – review and editing (equal). **Kai Wang:** Funding acquisition (equal); writing – review and editing (equal). **Ping Huang:** Investigation (equal); visualization (equal); writing – review and editing (equal). **Jiana Shi:** Conceptualization (equal); project administration (equal); writing – review and editing (equal).

## FUNDING INFORMATION

This work was supported by Outstanding young talents fund of Zhejiang traditional Chinese medicine (2022ZQ014), Sichuan Natural Science Foundation Program from Sichuan Provincial Department of Science and Technology (2023NSFSC0741), National college students innovation and entrepreneurship training program (202,310,632,039;202,310,632,104).

## CONFLICT OF INTEREST STATEMENT

All authors declare no conflict of interest.

## Supporting information


Figure S1.

Figure S2.

Table S1.
Table S2.
Table S3.
Table S4.
Table S5.
Table S6.
Table S7.
Table S8.
Table S9.
Table S10.
Table S11.

## Data Availability

The data sets of the present study are available from the corresponding author upon reasonable request.

## References

[1] Engstrand J , Nilsson H , Strömberg C , Jonas E , Freedman J . Colorectal cancer liver metastases—A population‐based study on incidence, management and survival. BMC Cancer. 2018;18(1):78. doi:10.1186/s12885-017-3925-x 29334918 PMC5769309

[2] Li R , Liu X , Huang X , et al. Single‐cell transcriptomic analysis deciphers heterogenous cancer stem‐like cells in colorectal cancer and their organ‐specific metastasis. Gut. 2024;73(3):470‐484. doi:10.1136/gutjnl-2023-330243 38050068 PMC10894846

[3] Tanaka A , Ogawa M , Zhou Y , et al. Proteogenomic characterization of primary colorectal cancer and metastatic progression identifies proteome‐based subtypes and signatures. Cell Rep. 2024;43(2):113810. doi:10.1016/j.celrep.2024.113810 38377004 PMC11288375

[4] Guo TA , Lai HY , Li C , et al. Plasma extracellular vesicle Long RNAs have potential as biomarkers in early detection of colorectal cancer. Front Oncol. 2022;12:829230. doi:10.3389/fonc.2022.829230 35480120 PMC9037372

[5] Chen HN , Shu Y , Liao F , et al. Genomic evolution and diverse models of systemic metastases in colorectal cancer. Gut. 2022;71(2):322‐332. doi:10.1136/gutjnl-2020-323703 33632712 PMC8762014

[6] Zhang Y , Song J , Zhao Z , et al. Single‐cell transcriptome analysis reveals tumor immune microenvironment heterogenicity and granulocytes enrichment in colorectal cancer liver metastases. Cancer Lett. 2020;470:84‐94. doi:10.1016/j.canlet.2019.10.016 31610266

[7] Liu X , Wang X , Yang Q , et al. Th17 cells secrete TWEAK to trigger epithelial‐mesenchymal transition and promote colorectal cancer liver metastasis. Cancer Res. 2024;84(8):1352‐1371. doi:10.1158/0008-5472.Can-23-2123 38335276

[8] Liu Y , Zhang Q , Xing B , et al. Immune phenotypic linkage between colorectal cancer and liver metastasis. Cancer Cell. 2022;40(4):424‐437. doi:10.1016/j.ccell.2022.02.013 35303421

[9] Cañellas‐Socias A , Sancho E , Batlle E . Mechanisms of metastatic colorectal cancer. Nat Rev Gastroenterol Hepatol. 2024;21:609‐625. doi:10.1038/s41575-024-00934-z 38806657

[10] Kong WS , Li JJ , Deng YQ , Ju HQ , Xu RH . Immunomodulatory molecules in colorectal cancer liver metastasis. Cancer Lett. 2024;598:217113. doi:10.1016/j.canlet.2024.217113 39009068

[11] Li X , Pan J , Liu T , et al. Novel TCF21(high) pericyte subpopulation promotes colorectal cancer metastasis by remodelling perivascular matrix. Gut. 2023;72(4):710‐721. doi:10.1136/gutjnl-2022-327913 36805487 PMC10086488

[12] Giguelay A , Turtoi E , Khelaf L , et al. The landscape of cancer‐associated fibroblasts in colorectal cancer liver metastases. Theranostics. 2022;12(17):7624‐7639. doi:10.7150/thno.72853 36438498 PMC9691344

[13] Wan R , Chen Y , Feng X , et al. Exercise potentially prevents colorectal cancer liver metastases by suppressing tumor epithelial cell stemness via RPS4X downregulation. Heliyon. 2024;10(5):e26604. doi:10.1016/j.heliyon.2024.e26604 38439884 PMC10909670

[14] Ganesh K , Basnet H , Kaygusuz Y , et al. L1CAM defines the regenerative origin of metastasis‐initiating cells in colorectal cancer. Nat Can. 2020;1(1):28‐45. doi:10.1038/s43018-019-0006-x PMC7351134 32656539

[15] Wang R , Li J , Zhou X , et al. Single‐cell genomic and transcriptomic landscapes of primary and metastatic colorectal cancer tumors. Genome Med. 2022;14(1):93. doi:10.1186/s13073-022-01093-z 35974387 PMC9380328

[16] Liu Z , Hu Y , Xie H , et al. Single‐cell chromatin accessibility analysis reveals the epigenetic basis and signature transcription factors for the molecular subtypes of colorectal cancers. Cancer Discov. 2024;14(6):1082‐1105. doi:10.1158/2159-8290.Cd-23-1445 38445965

[17] Cañellas‐Socias A , Cortina C , Hernando‐Momblona X , et al. Metastatic recurrence in colorectal cancer arises from residual EMP1(+) cells. Nature. 2022;611(7936):603‐613. doi:10.1038/s41586-022-05402-9 36352230 PMC7616986

[18] Wu Y , Yang S , Ma J , et al. Spatiotemporal immune landscape of colorectal cancer liver metastasis at single‐cell level. Cancer Discov. 2022;12(1):134‐153. doi:10.1158/2159-8290.Cd-21-0316 34417225

[19] Mo S , Tang P , Luo W , et al. Patient‐derived organoids from colorectal cancer with paired liver metastasis reveal tumor heterogeneity and predict response to chemotherapy. Adv Sci. 2022;9(31):e2204097. doi:10.1002/advs.202204097 PMC9631073 36058001

[20] Wang R , Mao Y , Wang W , et al. Systematic evaluation of colorectal cancer organoid system by single‐cell RNA‐Seq analysis. Genome Biol. 2022;23(1):106. doi:10.1186/s13059-022-02673-3 35484598 PMC9047329

[21] Che LH , Liu JW , Huo JP , et al. A single‐cell atlas of liver metastases of colorectal cancer reveals reprogramming of the tumor microenvironment in response to preoperative chemotherapy. Cell Discov. 2021;7(1):80. doi:10.1038/s41421-021-00312-y 34489408 PMC8421363

[22] Wang F , Long J , Li L , et al. Single‐cell and spatial transcriptome analysis reveals the cellular heterogeneity of liver metastatic colorectal cancer. Sci Adv. 2023;9(24):eadf5464. doi:10.1126/sciadv.adf5464 37327339 PMC10275599

[23] Ge LL , Wang ZC , Wei CJ , et al. Unraveling intratumoral complexity in metastatic dermatofibrosarcoma protuberans through single‐cell RNA sequencing analysis. Cancer Immunol Immunother. 2023;72(12):4415‐4429. doi:10.1007/s00262-023-03577-2 37938367 PMC10992304

[24] Fan C , Chen F , Chen Y , et al. irGSEA: the integration of single‐cell rank‐based gene set enrichment analysis. Brief Bioinform. 2024;25(4):bbae243. doi:10.1093/bib/bbae243 PMC11129768 38801700

[25] Barbie DA , Tamayo P , Boehm JS , et al. Systematic RNA interference reveals that oncogenic KRAS‐driven cancers require TBK1. Nature. 2009;462(7269):108‐112. doi:10.1038/nature08460 19847166 PMC2783335

[26] Hua X , Xu F , Shi W , et al. Prognostic significance of platelet‐to‐albumin ratio in patients with nasopharyngeal carcinoma receiving concurrent chemoradiotherapy: a retrospective study of 858 cases. BMC Cancer. 2024;24(1):762. doi:10.1186/s12885-024-12499-w 38918690 PMC11197365

[27] Aalen OO . Further results on the non‐parametric linear regression model in survival analysis. Stat Med. 1993;12(17):1569‐1588. doi:10.1002/sim.4780121705 8235179

[28] Ye Q , Yang X , Zheng S , et al. Low expression of moonlight gene ALAD is correlated with poor prognosis in hepatocellular carcinoma. Gene. 2022;825:146437. doi:10.1016/j.gene.2022.146437 35318110

[29] Efremova M , Vento‐Tormo M , Teichmann SA , Vento‐Tormo R . CellPhoneDB: inferring cell‐cell communication from combined expression of multi‐subunit ligand‐receptor complexes. Nat Protoc. 2020;15(4):1484‐1506. doi:10.1038/s41596-020-0292-x 32103204

[30] Xuan Z , Liu L , Zhang G , et al. Novel cell subtypes of SPP1 + S100P+, MS4A1‐SPP1 + S100P+ were key subpopulations in intrahepatic cholangiocarcinoma. Biochim Biophys Acta, Gen Subj. 2023;1867(9):130420. doi:10.1016/j.bbagen.2023.130420 37433400

[31] Jin T , Ding L , Chen J , et al. BUB1/KIF14 complex promotes anaplastic thyroid carcinoma progression by inducing chromosome instability. J Cell Mol Med. 2024;28(7):e18182. doi:10.1111/jcmm.18182 38498903 PMC10948175

[32] de Visser KE , Joyce JA . The evolving tumor microenvironment: from cancer initiation to metastatic outgrowth. Cancer Cell. 2023;41(3):374‐403. doi:10.1016/j.ccell.2023.02.016 36917948

[33] Wei C , Yang C , Wang S , et al. Crosstalk between cancer cells and tumor associated macrophages is required for mesenchymal circulating tumor cell‐mediated colorectal cancer metastasis. Mol Cancer. 2019;18(1):64. doi:10.1186/s12943-019-0976-4 30927925 PMC6441214

[34] Shin AE , Giancotti FG , Rustgi AK . Metastatic colorectal cancer: mechanisms and emerging therapeutics. Trends Pharmacol Sci. 2023;44(4):222‐236. doi:10.1016/j.tips.2023.01.003 36828759 PMC10365888

[35] Li Y , He X , Li Q , et al. EV‐origin: enumerating the tissue‐cellular origin of circulating extracellular vesicles using exLR profile. Comput Struct Biotechnol J. 2020;18:2851‐2859. doi:10.1016/j.csbj.2020.10.002 33133426 PMC7588739

[36] Xiu B , Chi Y , Liu L , et al. LINC02273 drives breast cancer metastasis by epigenetically increasing AGR2 transcription. Mol Cancer. 2019;18(1):187. doi:10.1186/s12943-019-1115-y 31856843 PMC6921600

[37] Zhang C , Qiao H , Guo W , et al. CD100‐plexin‐B1 induces epithelial‐mesenchymal transition of head and neck squamous cell carcinoma and promotes metastasis. Cancer Lett. 2019;455:1‐13. doi:10.1016/j.canlet.2019.04.013 30981760

[38] Chen WG , Sun J , Shen WW , et al. Sema4D expression and secretion are increased by HIF‐1α and inhibit osteogenesis in bone metastases of lung cancer. Clin Exp Metastasis. 2019;36(1):39‐56. doi:10.1007/s10585-018-9951-5 30617444

[39] Zuazo‐Gaztelu I , Pàez‐Ribes M , Carrasco P , et al. Antitumor effects of anti‐Semaphorin 4D antibody unravel a novel Proinvasive mechanism of vascular‐targeting agents. Cancer Res. 2019;79(20):5328‐5341. doi:10.1158/0008-5472.Can-18-3436 31239269 PMC7611261

[40] Ikeya T , Maeda K , Nagahara H , Shibutani M , Iseki Y , Hirakawa K . The combined expression of Semaphorin4D and PlexinB1 predicts disease recurrence in colorectal cancer. BMC Cancer. 2016;16:525. doi:10.1186/s12885-016-2577-6 27456345 PMC4960918

