# Supplementary figures and images for: PLXNB1/SEMA4D signals mediate interactions between malignant epithelial and immune cells to promote colorectal cancer liver metastasis
Source: J Cell Mol Med. 2024 Oct 23;28(20):e70142. doi: 10.1111/jcmm.70142 (PMC11499074; doi:10.1111/jcmm.70142)

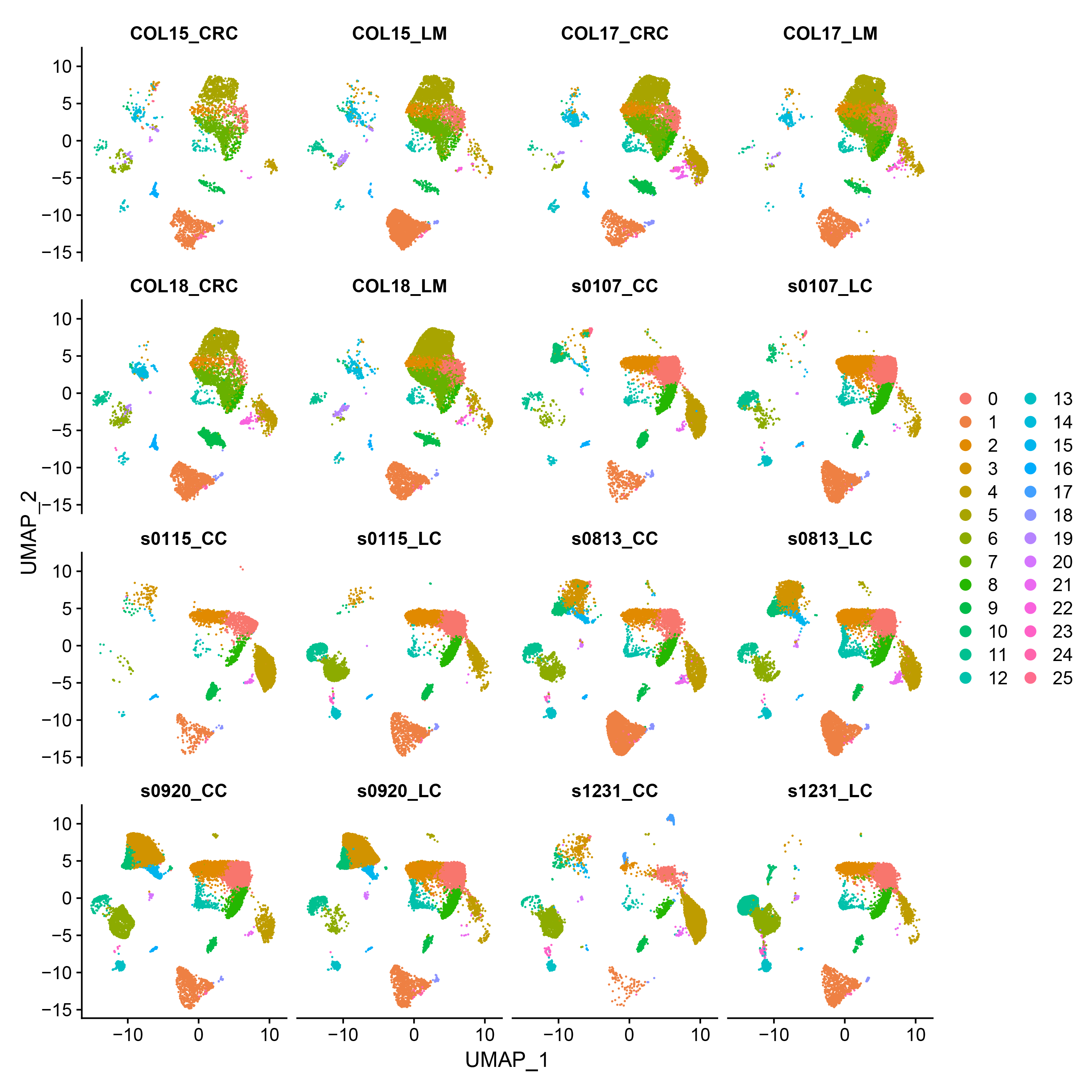

Supplement: Supplementary file 1 — Figure S1. [file JCMM-28-e70142-s002.tif]

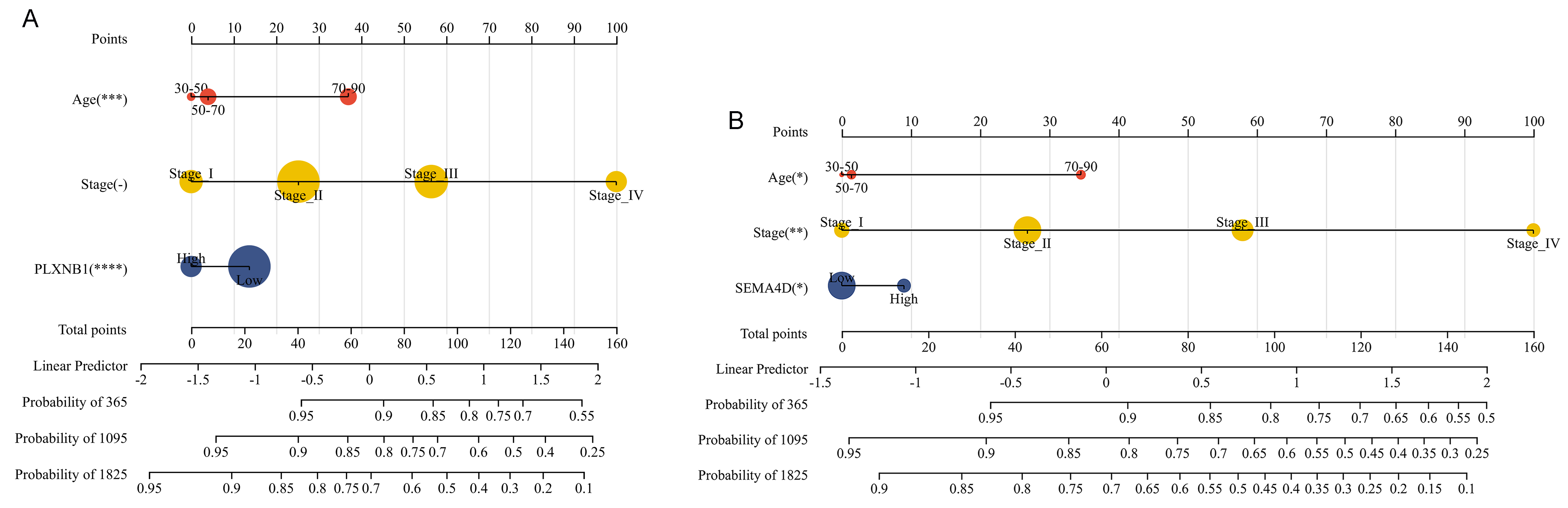

Supplement: Supplementary file 2 — Figure S2. [file JCMM-28-e70142-s001.tif]
